# New diagnosis of mother-to-child transmission of HIV in 8 Latin-American countries during 2018

**DOI:** 10.1186/s12879-022-07311-8

**Published:** 2022-04-07

**Authors:** Alicia Hernanz-Lobo, Beatriz Ruiz Saez, Itziar Carrasco García, Greta Mino-Leon, Julio Juárez, Noris Pavía Ruz, Dora Estripeaut, María de los Ángeles Pérez, Karen Erazo, Luis Guillermo Castaneda Villatoro, Oscar Porras, Luis Manuel Prieto Tato, María Luisa Navarro Gómez, on behalf of the CYTED PLANTAIDS group

**Affiliations:** 1 Instituto de Investigación Sanitaria Gregorio Marañón, Madrid, Spain; 2 Department of Paediatrics, Hospital Reina Sofía, Córdoba, Spain; 3 Centro de Investigación Biomédica en Red de Enfermedades Infecciosas, Instituto de Salud Carlos III, Madrid, Spain; 4 Department of Paediatrics, Hospital del Niño Dr. Francisco de Icaza Bustamante, Guayaquil, Ecuador; 5 CYTED (Ibero-American Programme of Science and Technology for Development), https://www.cyted.org/es/plantaids; 6 Department of Paediatrics, Hospital Roosevelt, Guatemala City, Guatemala; 7 Paediatric HIV/AIDS Clinic, Facultad de Medicina, UNAM/HGM, Universidad Nacional Autónoma de México, Mexico City, México; 8 Department of Paediatrics, Hospital del Niño Dr. José Renán Esquivel, Panama City, Panama; 9 Sistema Nacional de Investigación (SNI) de la Secretaría Nacional de Ciencia, Tecnología e Innovación (SENACYT), Panama City, Panama; 10 Department of Paediatrics, Hospital Infantil de Nicaragua, Managua, Nicaragua; 11 Department of Paediatrics, Hospital Dr. Mario Catarino Rivas, San Pedro Sula, Honduras; 12 Department of Paediatrics, Hospital Nacional de Niños Benjamin Bloom, San Salvador, El Salvador; 13 Department of Paediatrics, Hospital Nacional de Niños Dr. Carlos Sáenz Herrera, San José, Costa Rica; 14 Department of Paediatrics, Hospital Doce de Octubre, Madrid, Spain; 15 Department of Paediatrics, Hospital General Universitario Gregorio Marañón, Madrid, Spain; 16 Department of Paediatrics, Complutense University of Madrid, Madrid, Spain

**Keywords:** HIV, Infectious disease transmission, vertical, Pregnant women

## Abstract

**Background:**

Important prevention efforts have led to a reduction in mother-to-child transmission of HIV (MTCT) globally. However, new cases of paediatric HIV infections still occur. Early diagnosis of new HIV infections is essential to start an appropriate antiretroviral treatment to avoid childhood morbidity and mortality related to infection. The aim of this study was to describe the new cases of MTCT in Latin-American referral hospitals.

**Methods:**

A retrospective, multicentre and descriptive study of the new cases of MTCT diagnosed during 2018 in 13 referral hospitals from 8 Latin-American countries (Costa Rica, Ecuador, El Salvador, Guatemala, Honduras, Mexico, Nicaragua, and Panama) belonging to PLANTAIDS (Paediatric Network for Prevention, Early Detection and Treatment of HIV in Children), was conducted. PLANTAIDS is included in CYTED (Ibero-American Programme of Science and Technology for Development).

**Results:**

Eighty-one children (40.7% males) were included, median age at diagnosis of 2.33 years (IQR:0.7–4.7). Less than 3% of women knew their HIV diagnosis before pregnancy. More than 80% of them were diagnosed after delivery, 8.7% during pregnancy, and 2.9% at delivery. Only one patient underwent antiretroviral therapy (ART) prior to pregnancy. At diagnosis, 50.0% of the children presented with an advanced stage of disease (stage C following the current CDC classification for HIV infection), and 34.4% had less than 15% CD4^+^ cells/mm^3^. The time elapsed between delivery and the maternal diagnosis was correlated with the age of children at diagnosis, ρ = 0.760, *p* < 0.001. Younger age at diagnosis (*p* = 0.03), a smaller number of previous hospitalizations (*p* < 0.01), and better immunovirological status (*p* < 0.01) were found in children whose mothers knew their HIV status at delivery, compared to mothers who were not aware of it.

**Conclusions:**

Although MTCT in Latin America has declined in recent years, our series shows there are still cases that indicate some failures in prevention, being a critical point to improve an earlier diagnosis of pregnant women. Half of the children were diagnosed in an advanced stage of disease and the delay in maternal diagnosis entailed a worse clinical and immunological child’ prognosis.

## Introduction

Ending the HIV (human immunodeficiency virus) pandemic is part of the Sustainable Development Goals of the 2030 Agenda signed by the United Nations [1]. However, the number of new HIV infections is growing in Latin America. Preventing mother-to-child transmission of HIV (MTCT) is key to a reduction of the global incidence of new HIV infections and to avoiding paediatric HIV-related morbidity and mortality.

MTCT can occur in three moments: during pregnancy, at birth, or in the postpartum period and through breastfeeding. Preventing transmission at these stages is crucial. According to the latest Pan-American Health Organization report in 2019, MTCT in Latin America has been reduced in the last decade thanks to the measures recommended in the international clinical guidelines. The main interventions are antiretroviral therapy (ART) during pregnancy and at neonatal period, caesarean section (CS) before labour and rupture of membranes, in case of detectable viral load; and the contraindication of breastfeeding for women living with HIV in settings with no increased risk of death from malnutrition, diarrhoea or pneumonia if the infants are not breastfed [2].

The World Health Organization estimated that in 2010, 21,000 pregnant women were HIV positive in Latin America, and only 10,600 (50.5%) received ART; this year, 3400 children under 15 years old were diagnosed with HIV. In 2017, there were also 21,000 HIV-positive women, of whom 15,300 (72.9%) were on ART. The number of new paediatric diagnoses of HIV decreased to 2400 (29%) mainly related to the implementation of MTCT prevention measures [3].

El Salvador, Nicaragua, and Panama have a MTCT rate between 2 and 5%; Costa Rica, Ecuador, Guatemala, Honduras, and Mexico have made progress over the last 10 years and are also moving towards the elimination of MTCT, although their MTCT is over 5% [3]. Nevertheless, new cases of HIV infections in children still occur. These cases represent lost opportunities for prevention and a detailed analysis of each case may help to identify gaps and aid as an important tool to improve diagnostic and therapeutic algorithms [4]. Early knowledge of HIV infection leads to timely initiation of ART and reduces morbidity and mortality rates [5].

The main objective of the study is to describe the epidemiological, clinical, and analytical characteristics of new MTCT diagnoses in 2018 in CYTED’s (Ibero-American Programme of Science and Technology for Development) PLANTAIDS Network (Paediatric Network for Prevention, Early Detection and Treatment of HIV in Children).

The secondary objective is the analysis of the failures in the early prevention of MTCT to raise awareness for future design of strategies to reduce vertical transmission in Latin-American countries included in the cohort.

## Material and methods

### Design

**Fig. 1:**
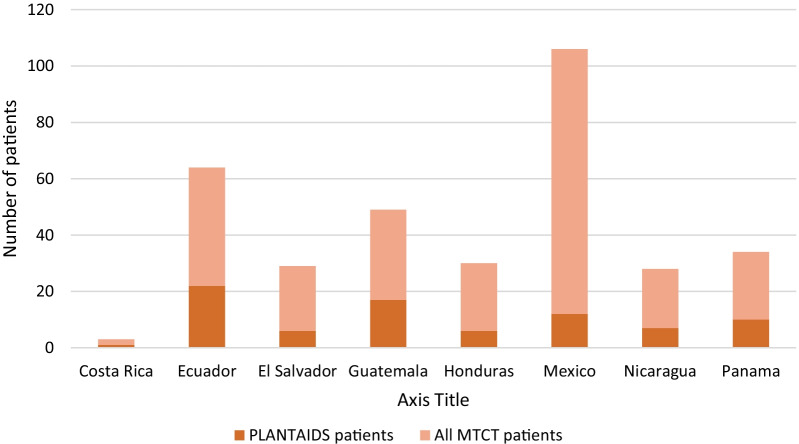
MTCT patients diagnosed in 2018; PLANTAIDS: Paediatric Network for Prevention, Early Detection and Treatment of HIV in Children. MTCT: mother-to-child-transmission of HIV (human immunodeficiency virus). [2] Source: UNAIDS 2018 estimates

Retrospective, multicentre and descriptive study of the new cases of MTCT diagnosed during 2018 in 13 referral hospitals from 8 Latin-American countries (Costa Rica, Ecuador, El Salvador, Guatemala, Honduras, Mexico, Nicaragua and Panama) belonging to CYTED’s PLANTAIDS Network. The number of cases and the percentage they represent from the total cases in each country is detailed in Fig. 1.

CYTED was created in 1984 by the governments of the Ibero-American countries to promote cooperation in science, technology, and innovation for the development of Ibero-America. PLANTAIDS network is one of the 28,000 projects undertaken through CYTED. It includes clinical researchers involved in the care of children with HIV infection from 8 Latin-American countries (Costa Rica, Ecuador, El Salvador, Guatemala, Honduras, Mexico, Nicaragua, and Panama), the Spanish paediatric HIV network, the French National Institute of Health and Medical Research and the Italo-Latin American Institute.

### Ethical approval and consent to participate

The study has been approved by the Ethics and Clinical Research Committee of the 12 de Octubre Hospital (Madrid, Spain), and subsequently ratified by all the Research Ethics Committees of the centres involved in the study. Data use was made in compliance with the provisions of each country’s regulations and the current law in Spain regarding data protection and Organic Law 3/2018 of 5th December for the Protection of Personal Data. The study complies with the rules of the Helsinki Declaration, and informed consent was obtained from all subjects and/or their legal guardian(s).

### Variables

Data were collected using Research Electronic Data Capture tools, hosted at Fundación de Investigación Biomédica 12 de Octubre (Madrid, Spain). In total, 113 children aged 0–18 years old, with new HIV diagnosis during 2018, were included in the database. Seventeen of them had a sexually transmitted infection. In another 14, the way of transmission was unknown, and one child was infected through breastfeeding, fed by a woman other than her mother. No other mode of transmission was described (blood transfusion or injecting drug users). All these patients were excluded, so finally, 81 mother–child pairs with evidence of MTCT were included in the study. Epidemiological characteristics, family history and prenatal data related to pregnancy follow-up, maternal HIV diagnosis, ART, immunovirological classification during pregnancy, and other maternal co-infections, were included. Whether or not an HIV test had been performed during pregnancy was not recorded. Data about delivery, gestational age, ART taken during delivery, and anthropometrical data of the newborn were also collected. Regarding the newborn and child follow-up, we included the following data: type of lactation (breastfeeding or formula feeding), antiretroviral (ARV) prophylaxis taken during the perinatal period, time of HIV diagnosis, symptoms prior to diagnosis, history of coinfections, and the Centers for Diseases Control and Prevention (CDC) HIV immunovirological classification.

### Definitions

Mother-to-child-transmission of HIV was considered when HIV was transmitted vertically or through breastfeeding given by the biological mother of the child.

Adequate prenatal care, in accordance with local guidelines, was considered when at least 4 prenatal consultations had been carried out.

Adherence to ART: good adherence is considered when the patient takes more than 95% of the medication doses, as reported by themselves.

First level medical centre: health care centre, defined as an outpatient facility.

Second level medical centre: regional or rural hospital.

Third level medical centre: specialised hospital.

### Statistical analysis

Qualitative variables were expressed as absolute frequencies and percentages, while quantitative variables were expressed as median and interquartile ranges (IQR).

The association between different clinical-analytical variables was compared using the Chi-square test in the case of categorical variables. Fisher’s exact test was conducted to compare categorical variables when at least one expected cell count was less than five. Mann Whitney's *U* test was used in the case of quantitative variables. In addition, the quantitative variables were categorized into ranges after their graphic exploration.

Interaction between qualitative variables was evaluated by stratified analysis, and the risk was quantified by odds ratio (OR) and its 95% confidence intervals (CI95%), following individual bivariate tests. Spearman rho (ρ) was used to assess the relationship between the time of maternal diagnosis and the age of children at diagnosis.

The statistical analysis was carried out using SPSS software for Windows (version 25). Statistical significance was set at *p* value < 0.05.

## Results

Eighty-one paediatric patients (40.7% males) diagnosed with MTCT were included in PLANTAIDS Network during 2018. The median age at diagnosis was 2.3 years old (IQR: 0.7–4.7). Twenty-two children were from Ecuador, seventeen from Guatemala, twelve from Mexico, ten from Panama, seven from Nicaragua, six from El Salvador, six from Honduras, and one from Costa Rica. The most prevalent ethnic group was Hispanic (70 children, 86.4%), there were 9 indigenous children, and 2 black patients. Eighty families provided data about the place of residence: 42 lived in rural areas (52.5%), and 38 in urban settings (47.5%). Table 1 summarizes family history and epidemiological background. One child was abandoned and found on a public road at 9 months old, already infected. We do not have any data of his biological parents. Two thirds of patients had siblings, 3.0% of whom were known to be HIV-positive. As for the place of birth, 10 (12.3%) were at home, 3 (3.8%) in first-level centres, 40 (50.0%) in second-level centres, and 27 (33.8%) in-third level centres. There were 49 (61.3%) vaginal deliveries and 31 (38.8%) CS.

**Table 1:** Family and epidemiological background

	Mothers (N = 80)	Fathers (N = 67)
HIV positive (%)*	80/80 (100)	53/61 (86.9)
Median age at delivery (years)	26.0 (21.3–33.0)	No data
Educational level (%)*		
None	12/68 (17.6)	14/55 (25.5)
Primary education	36/68 (52.9)	23/55 (41.8)
Secondary education	16/68 (23.5)	13/55 (23.6)
University level	4/68 (5.9)	5/55 (9.1)
Alive (%)*	64/79 (79.0)	53/67 (79.1)
Transmission route (%)*	Sexual 73/74 (98.6)	No data (n/d)
	Vertical 1/74 (1.4)	

*HIV* human immunodeficiency virus

*The denominator in each cell varies depending on the data available

At diagnosis, 49.3% of the children presented with an advanced stage of disease (stage C, following the current CDC classification for HIV infection [6]), and 34.4% had less than 15% CD4^+^ cells/mm3. Regarding symptoms prior to diagnosis, more than one out of two reported recurrent bacterial infections.

Overall, 2/69 women (2.9%) knew about their HIV diagnosis before pregnancy, 6 (8.7%) were diagnosed during pregnancy, 2 (2.9%) at delivery, and 59 (85.5%) at some point after delivery. There were no significant differences in women's literacy levels or place of residence in terms of the time of HIV diagnosis. In total, 47/61 women (77.0%) had adequate prenatal care, with a median of 5 antenatal medical consultations (IQR: 3–6). Only one patient received ART prior to pregnancy and she always maintained an undetectable viral load, although with a low CD4^+^ level. This mother/child couple was lost to follow-up after a first negative polymerase chain reaction (PCR) during the neonatal period. The child received breastfeeding for 24 months and could not be diagnosed until 4 years and 11 months of age in a state of significant clinical and immunological deterioration.

Regarding maternal immune status in the third trimester of pregnancy, total CD4^+^ lymphocyte count (cells/µl) data were obtained from 7 women: 2 presented < 200 CD4^+^, 4 between 200 and 300, and 1 > 750. Table 2 shows data of the 5 women with known viral load at delivery and the diagnosis of their newborns.

**Table 2:** Data of the five women with known viral load at delivery

	Patient 1	Patient 2	Patient 3	Patient 4	Patient 5
*Mother*					
Age (years)	27	33	26	34	35
Timing of HIV diagnosis	Delivery	3rd trimester	Pregestational	Delivery	Pregestational
CD4 count in third trimester (cel/µl)	200–300	200–300	> 750	< 200	< 200
Viral load at childbirth (copies/ml)	10,000–100,000	10,000–100,000	< 50	> 100,000	< 50
Gestational age at birth (weeks)	37	37	37	37	38
Type of delivery	Vaginal	CS	CS	CS	CS
ART before pregnancy	No	No	No	No	Yes (ZDV + 3TC + EFV)
ARV prophylaxis at delivery	No	n/d	n/d	n/d	ZDV iv
Child					
Birth weight (grams)	2000–4000	2000–4000	2000–4000	2000–4000	2000–4000
Type of ARV prophylaxis in the newborn	ZDV + 3TC + RTG	ZDV + 3TC	3TC	n/d	ZDV
Maternal breastfeeding	No	No	No	n/d	n/d
Age at diagnosis (months)	0.5	2	3	10	59
Viral load at diagnosis (copies/ml)	10,000–100,000	10,000–100,000	10,000–100,000	> 100,000	> 100,000
CD4 count at diagnosis (cel/µl)	> 750	> 750	> 750	> 750	> 750
CDC clinical classification of HIV at diagnosis	B	N	A	A	C

*HIV* human immunodeficiency virus, *CS* cesarean section, *ART* antiretroviral therapy, *ZDV* zidovudine, *3TC* lamivudine, *EFV* efavirenz, *ARV* antiretroviral prophylaxis, *n/d* no data available, *RTG* raltegravir, *CDC* Centers for Diseases Control and Prevention

Table 3 compares the characteristics of mothers who knew their diagnosis at delivery with those who were not aware of it, as well as their children’s. We could only confirm that 3/10 women whose HIV diagnosis was known at the time of delivery received ART during delivery: 2 with intravenous (iv) zidovudine (ZDV), and 1 patient with her usual ART, with poor adherence. The only woman who underwent ART before pregnancy was diagnosed 2 years before and started treatment two months before pregnancy with ZDV, lamivudine (3TC), and efavirenz (EFV), with adequate adherence. In the 14th week of pregnancy, due to EFV toxicity, it was replaced by nevirapine (NVP). She received intrapartum ZDV.

**Table 3:** Comparison between women with and without known diagnosis at delivery

	All* N = 69**	Women with known diagnosis at delivery N = 10**	Women without known diagnosis at delivery N = 59**	*p*	OR (95% CI)
*Pregnancy and delivery*					
Mother’s age at pregnancy (years)	26 (22.0–31.8)	21.5 (18.3–35.0)	26.0 (22.0–33.0)	0.99	
Prenatal care	47/61 (77.0)	9/10 (90)	38/51 (74.5)	0.43	
Delivery in a third level medical centre	23/69 (33.3)	5/10 (50)	18/59 (30.5)	0.26	
Gestational age (weeks)	38 (37–40)	38 (37–38)	38 (37–40)	0.05	
Caesarean delivery	27/68 (39.7)	9/9 (100.0)	18/59 (30.5)	< 0.01	3.3 (2.2–4.8)
Prematurity (< 37 weeks)	7/66 (10.6)	3/10 (30)	4/56 (7.1)	0.07	
Intrapartum ARV prophylaxis	3/64 (4.7)	3/6 (50.0)	0/58 (0.0)	< 0.01	
Invasive procedures	2/60 (3.3)	0/9 (0.0)	2/51 (3.9)	1.00	
Neonatal period					
ARV newborn prophylaxis	10/69 (14.5)	8/10 (80.0)	2/59 (3.4)	< 0.01	26.2 (6.6–104.4)
Duration of prophylaxis (median) (weeks)	0 (0.0–0.4)	4 (4.0–4.0)	0	< 0.01	
Cotrimoxazol prophylaxis	9/59 (15.3)	7/9 (77.8)	2/50 (4.0)	< 0.01	22.3 (5.6–89.0)
Artificial formula feeding	10/68 (14.7)	8/10 (80)	2/58 (3.4)	< 0.01	23.2 (5.7–93.8)
Diagnosis					
Number of children diagnosed later than their mothers	43/61 (70.5)	5/7 (71.4)	38/54 (70.4)	1.0	
Age at diagnosis (years)	2.3 (0.7–4.7)	0.3 (0.2–3.2)	2.4 (1.0–5.2)	0.03	
Previous hospitalizations	29/67 (43.3)	0/9 (0.0)	29/58 (50.0)	< 0.01	
Number of previous hospitalizations	1 (0–2)	0 (0–0)	1 (0–2)		
Low weight percentile (p ≤ 3)	23/66 (34.8)	2/9 (22.2)	21/57 (36.8)	0.17	
Low height percentile (p ≤ 3)	20/66 (30.3)	3/9 (33.3)	17/57 (29.8)	0.73	
Stage 1 following CDC immunological classification***	18/59 (30.5)	4/7 (57.1)	14/52 (26.9)	0.18	
Stage N/A following CDC clinical classification***	23/67 (34.3)	8/10 (80)	15/57 (26.3)	< 0.01	3.0 (1.8–5.2)
CD4 < 500 cel/µl	23/63 (36.5)	0/9 (0.0)	23/54 (42.6)	0.02	
CD4 < 15%	19/52 (36.5)	2/7 (28.6)	17/45 (37.8)	1.0	
Viral load < 100,000 copies/ml	25/64 (39.1)	5/10 (50)	20/54 (37.0)	0.49	
Symptoms at diagnosis					
Chronic diarrea	20/56 (35.7)	1/5 (20)	19/51 (37.3)	0.65	
Chronic parotitis	2/55 (3.6)	0/5 (0.0)	2/50 (4.0)	1.0	
Adenopathies	33/58 (56.9)	2/6 (33.3)	31/52 (59.6)	0.39	
Hepatomegaly	29/57 (50.9)	3/7 (42.9)	26/50 (52.0)	0.71	
Splenomegaly	14/53 (26.4)	1/6 (16.7)	13/47 (27.7)	1.0	
Neurological symptoms	2/54 (3.7)	1/5 (20)	1/49 (2.0)	0.18	
Psychomotor delay	25/54 (46.3)	2/5 (40)	23/49 (46.9)	1.0	
Oportunistic infections	23/59 (39.0)	1/6 (33.3)	22/53 (41.5)	0.39	
Severe acute malnutrition	32/52 (61.5)	1/5 (20)	31/47 (66.0)	0.07	
Chronic neumopathy	4/54 (7.4)	0/5 (0.0)	4/49 (8.2)	1.0	
Chronic anemia	17/53 (32.1)	0/5 (0.0)	17/48 (35.42)	0.16	
Chronic thrombocytopenia	6/53 (11.3)	0/5 (0.0)	6/48 (12.5)	1.0	
Tuberculosis	10/50 (20)	1/9 (11.1)	9/41 (22.0)	1.0	

*OR* odds ratio, *CI* confidence interval, *ARV* antiretroviral, *CDC* Centers for Diseases Control and Prevention

^*^There were no available data on the HIV status at delivery of the other 12 mothers

^**^N (%) or median (IQR)

^***^As explained in [6]

Regarding the newborns of these ten women with known HIV diagnosis at delivery, 5 were treated with combination ARV prophylaxis after birth for at least 4 weeks [2 with ZDV + 3TC + NVP, 1 with ZDV + 3TC + raltegravir (RTG), 1 with ZDV + 3TC, and 1 with 3TC + NVP]. One child received ZDV + 3TC for less than 4 weeks. Another 2 received single-drug prophylaxis (1 with ZDV and 1 with 3TC). All of them reported good adherence to treatment. Of the remaining 2, one did not receive ARV prophylaxis because the mother refused the HIV diagnosis and treatment when she was diagnosed during the 2nd trimester of pregnancy; no data were available for the other one. Newborns from women with known diagnosis at delivery were more likely to receive ARV prophylaxis (p < 0.01, OR 26.2; CI95%: 6.6–104.4), cotrimoxazole prophylaxis (p < 0.01, OR 22.3; CI95%: 5.6–89.0), and artificial formula feeding (p < 0.01, OR 23.2; CI95%: 5.7–93.8) than newborns from women without known diagnosis at delivery.

Only 1 of the 10 newborns whose mothers knew their HIV diagnosis at birth was diagnosed within the first month of life. Regarding the others, two infants were diagnosed at 2 months, and four between 3 and 5 months of age. The child of the woman who refused the HIV diagnosis could not be diagnosed until the age of 2 years and 8 months. The toddler whose mother underwent ART before and during pregnancy was diagnosed at 4 years 11 months old. The last child was diagnosed at 9 years without any previous screening test. Only 4.9% of the 81 children in our series were diagnosed during the neonatal period.

**Fig. 2:**
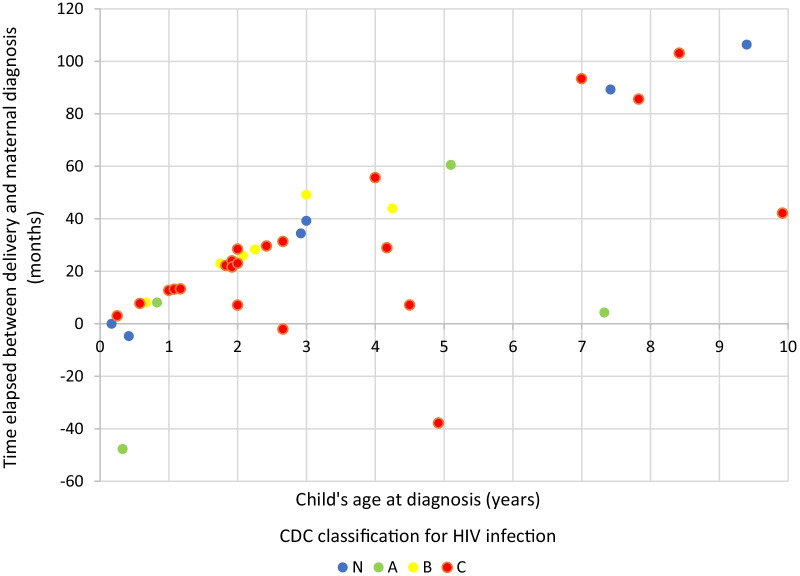
Correlation between the time of maternal diagnosis and child diagnosis. *CDC* Centers for Diseases Control and Prevention, *HIV* human immunodeficiency virus

The median delay for maternal diagnosis was 22.2 months after delivery (IQR: 5.8–34.4). The time elapsed between the delivery and the maternal diagnosis was correlated with the age of children at diagnosis, ρ = 0.760, *p* < 0.001 (Fig. 2). The correlation between these two variables is almost linear, although there are some outliers. One represents the pregnant woman who refused her HIV diagnosis, and for the other woman diagnosed 4 months after delivery and with late diagnosis of the child, there are no available data that could explain this delay. The lower the viral load and the higher the maternal CD4^+^ count during pregnancy, the lower the risk of transmission [7].

## Discussion

This series of new HIV diagnoses in children from Latin America shows important gaps and challenges in the prevention of MTCT and maternal/antenatal care.

In our series, most patients (77.0%) have undergone adequate prenatal care. Despite this, MTCT was not prevented. According to the results of our work, there is still a worrying problem of vertical transmission of HIV and screening in pregnant women. Universal HIV testing of all pregnant women has proven to be a cost-effective strategy to eliminate MTCT in both high- and low-endemic areas [8]. Ideally, this should be done before pregnancy, especially in women who have risk factors for HIV infection, so that during pregnancy, the infection will be under control. In our study, only two mothers knew about their HIV status before pregnancy, and only 11.6% of the women knew about their status before delivery. In our perspective, this is very alarming, since 80% of the transmissions reported in the study could not have been prevented as the serostatus of the mothers was unknown. In any case, HIV should always be included in the list of infectious pathologies to be screened in the first trimester and, in the event of proving negative, it has to be repeated in the third trimester, to be able to diagnose the new infections and those that were previously in window period [9]. The most important interventions in MTCT prevention have been the use of ART in pregnancy and the neonatal period, caesarean sections when a detectable viral load existed, and the contraindication of breastfeeding, when possible, all of which can only be applied if the woman’s serostatus is known before pregnancy.

The most typical way of transmission in women is heterosexual [5]. In fact, in our series, 98.6% of women reported a sexual transmission and only one reported vertical infection. In addition, 61 male partners of the women of the study were tested at the time of the child’s diagnosis and 86.9% of them were HIV-positive. In high endemic areas, an HIV test could be considered, not only for women with gestational desire or already pregnant, but also for sexual partners.

Half of the children whose mothers were unaware of their HIV diagnosis at the time of delivery were hospitalized for different reasons at least once before diagnosis, with their HIV status going unnoticed. Any contact of a child with a healthcare facility should be considered an opportunity to rule out HIV [10, 11]. This would prevent infected children from reaching the diagnosis in a state of clinical and immunological deterioration, negatively impacting the response to ART [12].

Early diagnosis during pregnancy decreases the risk of MTCT. In our series, there have been failures in the three stages of potential HIV diagnosis to prevent MTCT: before pregnancy, first and third trimester of pregnancy infectious screening, and after delivery. These are the so-called "missed opportunities" for screening maternal HIV infection. Only 2.5% of women knew their diagnosis before becoming pregnant, and 14.5% were aware of it at the time of delivery. For women whose serostatus is unknown at delivery, it is essential to perform a rapid test at that time.

On the other hand, out of the 10 patients who knew about their serostatus, antiretroviral treatment was used during delivery in only three of them. Therefore, our concern is why ART was not used during delivery in all patients who knew of their serostatus. The importance of treating all patients with a positive HIV test needs to be emphasized. In addition, out of the 10 children born to these 10 women, we know that only eight children received neonatal prophylaxis with ART, so the need for neonatal prophylaxis in newborns exposed to HIV during gestation and delivery also needs to be emphasized.

As shown in Fig. 2, the timing of maternal diagnosis is crucial for early diagnosis in children. In our series, there is a correlation between the time of maternal diagnosis and the age of the child at diagnosis: the later the mother is diagnosed, the longer the diagnosis of the child is delayed. The results of our work demonstrate, as expected, that delayed diagnosis from birth is associated with older age at diagnosis, more previous hospitalizations, and worse immune status. All these factors influence the progression of infection and disease in these patients. During breastfeeding, the risk of HIV transmission without any prophylactic measure amounts to 20–40% [7, 13–16]. In Latin America, where formula feeding and access to safe water are guaranteed in most of the territory, breastfeeding is not recommended in case of mother's HIV, regardless of the mother’s immunovirological status [17–23]. Despite this and given that most women did not know their HIV-positive status at delivery, in our series, 85.3% of children were breastfed. MTCT prevention measures should not be limited to gestation and should continue during breastfeeding, in case formula feeding is not possible [24]. Once breastfeeding is complete, children should be tested again [25]. Adherence to ART in childhood depends on socioeconomic factors and varies between regions [26, 27].

In our study, one woman refused the diagnosis and, in some other cases, after delivery, women with their children never returned for consultation, and the babies could not be adequately followed up, returning for consultation only when the child was already at an advanced stage of illness. To avoid these cases, financial and human resources need to be allocated to MTCT prevention and to minimise these follow-up losses [28, 29]. There is still a significant stigma surrounding the HIV pandemic, which makes it difficult to adequately follow-up patients, especially in low- and middle-income countries [30]. MTCT prevention has been remarkable in recent years with the implementation of the indicated measures; however, the picture of MTCT is still worrisome, making the intervention and insistence of health professionals essential.

Our study has several limitations. On the one hand, it is retrospective, with the consequent limitations. On the other hand, whether or not an HIV test had been performed during pregnancy was not recorded. Therefore, it was not possible to know the percentage of women who could have been diagnosed during pregnancy, or the percentage of those getting infected during breastfeeding. We considered 81 mother–child pairs of MTCT, but in the case of the 9-month-old child found on the road, the HIV status of the biological mother could not be confirmed, as we do not have any data on her. We assumed the MTCT, given the child’s young age. Logistically, it should be noted that, in many centres, there is no possibility of performing a CD8^+^ lymphocyte count. Moreover, as this is a multicentre study involving centres and researchers from different countries, there is variability in their respective clinical protocols and in the way clinical variables are collected, making it difficult to homogenise the data.

## Conclusions

MTCT in Latin America has globally declined in recent years. However, there are still cases that indicate some failures in prevention. It is essential to know the HIV status of all pregnant women. In our series, half of the children were diagnosed in an advanced stage of disease and the delay in maternal diagnosis worsened the clinical and immunological prognosis of the child.

Those women whose HIV diagnosis is known prior to pregnancy, or who are diagnosed during pregnancy, should receive ART and should be closely monitored to ensure early detection of possible risk factors for MTCT and avoid transmission. Mother and child should be followed up until infection in the child is ruled out in accordance with current protocols in each country.

Reviewing the medical history of any child accessing the health system should not be forgotten. If the mother or child have not been screened for HIV, a serology should be requested. If the child is finally diagnosed with HIV infection, the reason for the failure to prevent MTCT should be analysed, and ART started immediately.

## Data Availability

The datasets used and/or analysed during the current study are available from the corresponding author on reasonable request.

## Abbreviations

ART: Antiretroviral therapy
ARV: Antiretroviral
CDC: Centers for Diseases Control and Prevention
CI: Confidence interval
CS: Caesarean section
CYTED: Ibero-American Programme of Science and Technology for Development
EFV: Efavirenz
HIV: Human immunodeficiency virus
IQR: Interquartile range
MTCT: Mother-to-child-transmission of HIV
n/d: No data
NVP: Nevirapine
OR: Odds ratio
PCR: Polymerase chain reaction
PLANTAIDS: Paediatric Network for Prevention, Early Detection and Treatment of HIV in Children
RTG: Raltegravir
ZDV: Zidovudine
3TC: Lamivudine
